# Design of a Low-Cost Auto-Ranging Electrometer for Current-Mode Dosimetry

**DOI:** 10.3390/s26196279

**Published:** 2026-10-03

**Authors:** Marko Andjelković, Goran S. Ristić

**Affiliations:** 1 IHP— Leibniz Institute for High Performance Microelectronics, Im Technologiepark 25, 15236 Frankfurt (Oder), Germany; 2 Faculty of Electronic Engineering, University of Niš, Aleksandra Medvedeva 4, 18000 Niš, Serbia; goran.ristic@elfak.ni.ac.rs

**Keywords:** auto-ranging electrometer, low current measurement, current-mode gamma dosimetry, PIN photodiodes

## Abstract

This paper presents a low-cost auto-ranging electrometer for current-mode dosimetric applications. The proposed electrometer is based on a variable gain transimpedance amplifier, and has been realized with commercially available components. It can operate as a stand-alone unit with an integrated display for real-time measurement visualization, or can be interfaced to a personal computer (PC) over USB or RS-232 connections, allowing real-time visualization and data storage using custom-made PC application software. The electrometer is capable of measuring direct currents from 50 pA to 10 µA in three sensitivity ranges, with relative error less than 2.5%, and response time of approximately 0.2 s in each range. The performance of the proposed electrometer is comparable with custom-made electrometers reported in the literature, as well as with several standard commercial dosimetric electrometers. The electrometer has been validated experimentally for measuring the direct current of four commercial PIN photodiodes under gamma irradiation from a Co-60 source. The results have shown that the realized electrometer, combined with the investigated PIN photodiodes, can serve as a low-cost current-mode dosimeter for measuring the gamma radiation dose rates from 0.9 Gy/h to 34 Gy/h, and absorbed doses at least up to 33 Gy.

## 1. Introduction

Accurate measurement of ionizing radiation is essential in a wide range of applications, such as medical diagnostics and radiotherapy, radiation protection, environmental monitoring, high-energy physics experiments, space missions, and industrial radiation processing. Depending on the application requirements (e.g., sensitivity, energy range, response time, dynamic range, and operational environment), different types of radiation sensors and readout techniques may be employed.

Based on the readout principle, radiation sensors can be categorized into voltage-mode, current-mode, and pulse-mode sensors. Voltage-mode sensors measure the accumulated dose in terms of radiation-induced change of voltage across the sensing element. Typical examples of voltage-mode radiation sensors include standard pMOS transistors [1,2], specially designed pMOS transistors with enhanced radiation sensitivity, known as Radiation Sensitive Field Effect Transistors (RADFETs) [3,4,5,6], VDMOS transistors [7,8], and floating-gate transistors [9,10,11]. Current-mode sensors measure the continuous current generated in the sensing element during radiation exposure. The current is proportional to radiation dose rate, while integrating the current over a predefined time interval gives the total dose. Common examples of current-mode sensors are diodes [12,13,14], optoelectronic devices (photodiodes, phototransistors, light-dependent resistors) [15,16,17,18,19,20,21], ionization chambers [22], and diamond detectors [23]. In contrast to current-mode sensors, pulse-mode sensors generate individual current pulses in response to incident radiation, where the pulse amplitude, width, and frequency provide the information on radiation intensity (dose rate and total dose). Sensors employed in current mode can also operate in pulse mode, but with different readout electronics.

The choice between voltage-, current-, and pulse-mode radiation sensing depends on the expected radiation intensity as well as the specific system and application require-ments. Voltage-mode sensors are primarily employed for low-power measurement of total dose, and they can usually detect radiation without applied voltage bias. Current-mode sensors are suitable for continuous measurement of dose rate in medium- and high-intensity radiation fields. On the other hand, pulse-mode sensors are preferred for low-intensity (low dose rate) radiation sensing because they detect and count individual radiation events. In this work, the focus is on current-mode dosimeters.

Owing to their continuous analog output, wide dynamic range, fast response, and relatively simple signal processing requirements, current-mode dosimeters are widely employed in applications that require real-time monitoring of radiation dose rates. In medical radiotherapy, they enable accurate beam monitoring and dose verification during treatment delivery. In industrial facilities, they are used for radiation-supported sterilization and material processing. In aerospace missions, they provide long-term monitoring of radiation exposure to spacecraft electronics and astronauts, helping to assess cumulative radiation effects and support adaptive system operation. Similarly, in nuclear research facilities and particle accelerators, they are used for beam diagnostics and radiation safety assurance. Environmental radiation monitoring systems employ current-mode dosimeters for continuous surveillance in and around radiation-controlled areas.

The performance of current-mode radiation dosimeters is determined by the sensitivity of the sensing element and the accuracy of the readout electronics. In general, the currents generated by current-mode radiation sensors can span from femtoampere to microampere ranges. Accurate measurement of such low currents over wide dynamic range requires specially designed electrometers. The basic principle of an electrometer operation is the conversion of input current into voltage, and subsequent measurement of that voltage. Two principal approaches for current-to-voltage conversion are the shunt-based approach and the feedback-based approach. For low-level current measurement, the feedback-based approach provides better performance regarding stability and precision, and it is thus widely used in dosimetric electrometers.

From an implementation perspective, current-mode readout systems are typically realized as mixed-signal systems comprising a low-noise analog signal conditioning circuitry and digital data processing unit. A wide variety of commercial instruments for low-level current measurement are available, but they are very expensive and lack adaptability for field use or integration into custom-built systems. This can represent a significant barrier for research laboratories and educational institutions seeking to perform accurate radiation measurements at low cost. Applications requiring cost-effective instrumentation therefore motivate the development of customized electrometer solutions. Numerous custom-designed electrometers have been reported in the literature [24,25,26,27,28,29,30,31,32,33,34,35,36]. These solutions are realized either using only commercial components [24,25,26,27,28,29,30,31,32] or with custom-designed Application Specific Integrated Circuits (ASICs) [33,34,35,36]. However, only a few of the reported solutions have been verified under real irradiation conditions.

In this work, we present a custom-designed auto-ranging electrometer based on transimpedance current-to-voltage conversion technique, and supporting direct current measurements from 50 pA to 10 µA, with relative error less than 2.5% and response time of 0.2 s. The key contribution is in experimental verification of the developed electrometer, and comparison with both custom-designed and commercial dosimetric electrometers. The proposed electrometer was developed exclusively with low-cost commercial components, and has been tested with four commercial PIN photodiodes under gamma irradiation from a Co-60 source. The solution presented in this work is a significant upgrade of our initial electrometer [26], which was designed as a modular system fully dependent on the personal computer (PC) for controlling its operation.

The rest of the paper is organized as follows. Section 2 describes the basics of the transimpedance current-to-voltage conversion. The architecture of the realized electrometer and the design of each functional unit are described in Section 3. Section 4 presents the results of electrometer calibration in a laboratory, experimental results for measuring radiation-induced current in PIN photodiodes, and technical comparison of proposed electrometer with commercial and custom-made electrometers. Finally, Section 5 concludes the paper.

## 2. Transimpedance Current-to-Voltage Conversion

The feedback-based current-to-voltage conversion relies on using a standard operational amplifier with negative feedback as a conversion element. It can be achieved in the form of linear conversion or logarithmic conversion. The linear conversion can be accomplished with a transimpedance amplifier (operational amplifier with resistive feedback) or with a current integrating amplifier (operational amplifier with capacitive feedback). The logarithmic conversion is performed using a logarithmic amplifier composed of an operational amplifier with an active element (diode or transistor) in the negative feedback. In the case of the transimpedance technique, the wide dynamic range (current measurement over wide range) cannot be accomplished with a single feedback resistor, but it is necessary to use multiple switchable feedback resistors. Similarly, the wide-range current integration is achieved with multiple switchable capacitors and fixed integration time, or with a single capacitor and variable integration time. On the other hand, logarithmic amplifiers provide wide range response in a single measurement range with only one feedback element, resulting in very fast response but with lower resolution compared to linear current-to-voltage conversion techniques.

**Figure 1:**
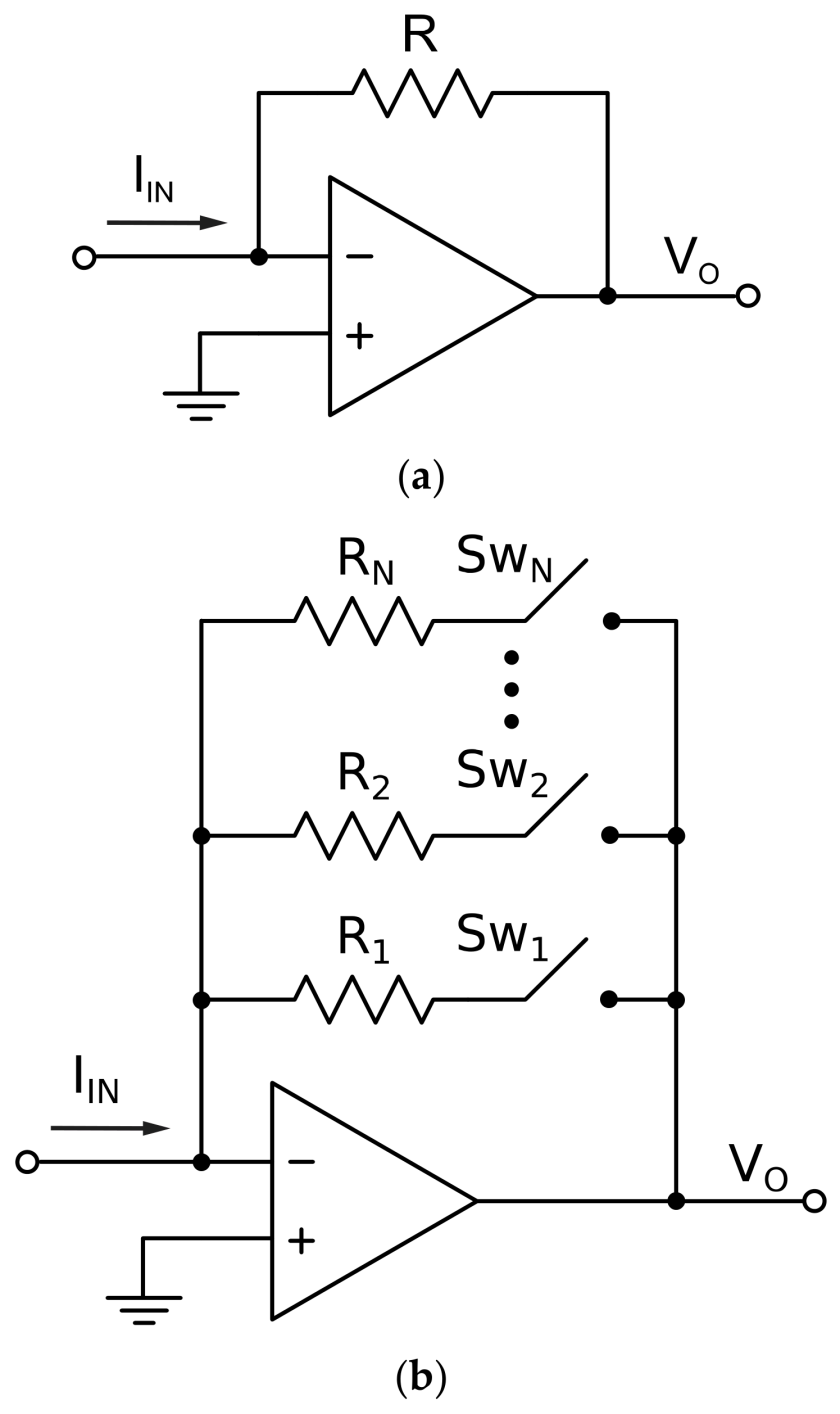
Transimpedance amplifier with: (**a**) fixed gain, (**b**) variable gain.

In this work, we have selected the transimpedance current-to-voltage conversion technique because it provides a faster response to changes in input current compared to the current integration technique. The basic configuration of a transimpedance amplifier is illustrated in Figure 1a. It consists of an operational amplifier and a resistor *R* connected between the inverting input of the operational amplifier and its output. The current *I_IN_* flowing into the inverting input of the operational amplifier is converted into the output voltage *V_O_* according to the transimpedance relation,

(1) $$V_{O}=-R\cdot \left(I_{IN}-I_{BIAS}\right)+V_{OFFSET}$$

where *I_BIAS_* is input bias current for the operational amplifier and *V_OFFSET_* is the offset voltage for the operational amplifier.

By choosing appropriate feedback resistor *R*, the input current can be transformed into a measurable voltage. The most suitable operational amplifiers for constructing the low-level current measurement circuits are those with very low input bias currents, very low offset voltage, and high input impedance. For low input bias current (in the range of fA), and low offset voltage (in the range of µV), the relation (1) can be simplified to express the output voltage in terms of input current and feedback resistor,

(2) $$V_{o}=-R\cdot I_{IN}$$

When it is required to achieve the current measurement in a wide dynamic range, use of a single feedback resistor is not sufficient. The solution is in the use of multiple switchable feedback resistors, as depicted in Figure 1b. In this configuration, higher resistances correspond to lower current ranges. Each resistor R_i_ defines a single measurement range according to relation (2), while the switching of resistors is accomplished with the corresponding analog switches Sw_i_ controlled by external logic. Choice of low leakage switch is essential for achieving the highest measurement accuracy. The switching of feedback resistor is managed by a dedicated controller that implements an auto-ranging algorithm for continuous monitoring of the output voltage.

## 3. Electrometer Design

**Figure 2:**
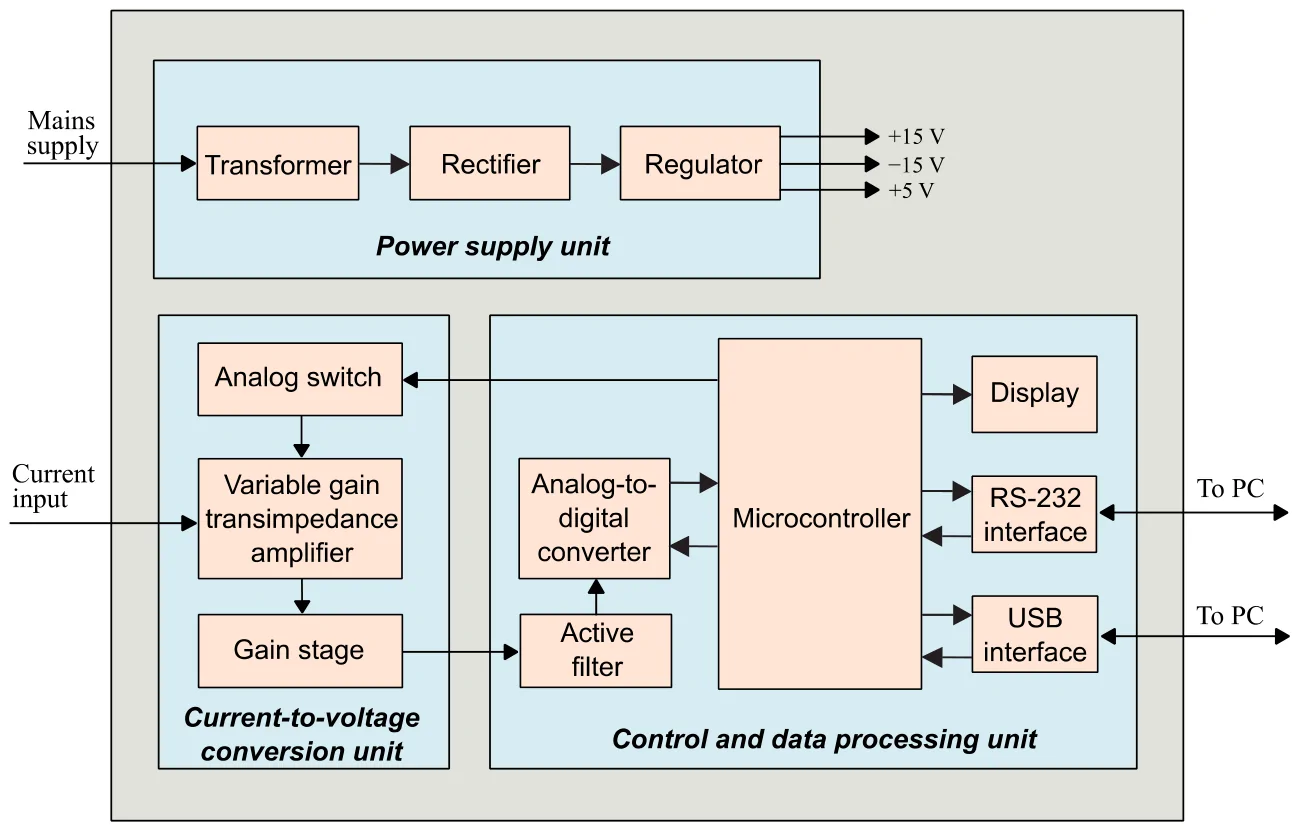
Proposed electrometer architecture.

Based on our preliminary design [26], an enhanced electrometer has been developed applying the variable gain transimpedance current-to-voltage conversion. In contrast to the previous solution [26], which was designed as a PC-based electrometer, the new version can operate as a stand-alone instrument with optional support for interfacing to a PC. Additionally, the response time has been decreased significantly through improving the firmware. The electrometer has been realized using only low-cost commercially available components. Figure 2 illustrates the block diagram of the electrometer composed of three main functional units: (i) Current-to-voltage conversion unit (CVCU), (ii) Control and data processing unit (CDPU), and (iii) Power supply unit (PSU). The architecture of each unit is described in the following.

To ensure fast response and smooth transition between the measurement ranges, the auto-ranging functionality has been implemented. This means that the electrometer can automatically detect the range to which the input current belongs, and accordingly adjust the appropriate settings for that range. Switching from one range to the other is achieved automatically, on the basis of the range switching algorithm defined by the firmware executed by the CDPU.

The proposed electrometer supports the direct current measurements in three sensitivity ranges, as given in Table 1.

### 3.1. Current-to-Voltage Conversion Unit (CVCU)

**Figure 3:**
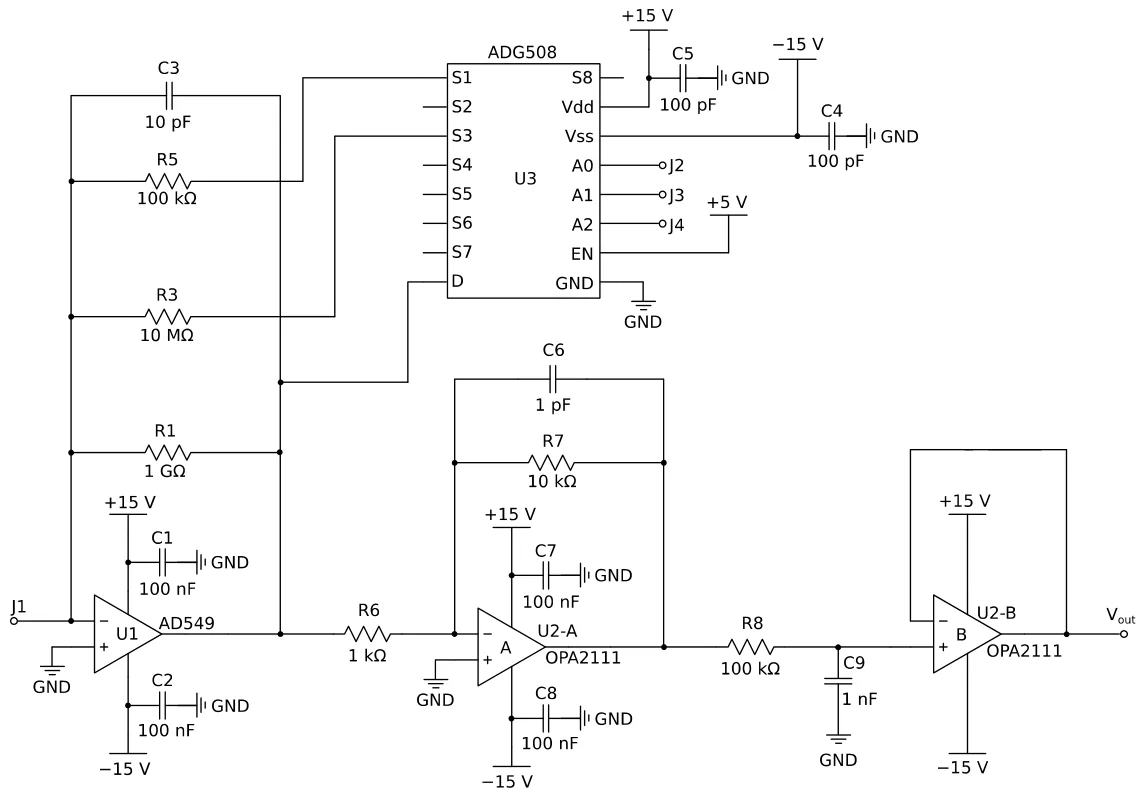
Current-to-voltage conversion unit.

The Current-to-Voltage Conversion Unit (CVCU) transforms the input current into a proportional voltage based on the principle of transimpedance gain, as defined by relation (2). The constitutive elements of the CVCU are as follows: a variable gain transimpedance amplifier, an inverting amplifier, and a gain switching logic. In Figure 3, the complete schematic of the CVCU is given.

The variable gain transimpedance amplifier consists of AD549J, an operational amplifier from Analog Devices (Wilmington, MA, USA), and three feedback resistors for gain setting (i.e., selection of measurement range), as shown in Figure 3. The AD549J is an electrometer-grade operational amplifier, featuring ultra-low input bias current (less than 250 fA), low input offset current (less than 3 fA), and very low input offset voltage (below 1 mV). Each resistor corresponds to one measurement range, and it determines the transimpedance gain between the input current I and the voltage V at the output of AD549J amplifier, according to the transimpedance relation (2). The resistor values and the corresponding current ranges are given in Table 2.

All implemented resistors are metal-film with 1% tolerance. The gain for Range 1 is defined by 1 GΩ resistor which is permanently fixed between the inverting input and the output of the AD549J operational amplifier. This resistor provides a fixed path for current flow and thus helps in minimizing the possible spikes that can occur during gain switching. For Ranges 2 and 3 the gain is determined by connecting an appropriate resistor in parallel with the 1 GΩ resistor, via the gain switching logic. By connecting any resistor in parallel with the 1 GΩ resistor, the effective resistance is approximately equal to the resistance connected to 1 GΩ (e.g., 10 MΩ in parallel with 1 GΩ gives an equivalent resistance of approximately 10 MΩ). A ceramic capacitor of 10 pF is inserted in the feedback of the AD549J operational amplifier to suppress the unwanted oscillations that might be induced during operation.

Gain switching is performed by ADG508, an 8-channel monolithic Complementary Metal Oxide Semiconductor (CMOS) analog switch from Analog Devices, featuring low leakage current (below 20 pA), low switching time (20 ns), and low on-resistance (280 Ω). The selected analog switch has four input pins for controlling the switch states (high logic level closes the switches, while low logic level opens the switches). The control of the switching is accomplished by the microcontroller in the CDPU.

For Range 1, the 1 GΩ feedback resistance converts the input current below 1 nA into a transimpedance output voltage from approximately 0 V to 1 V. For Range 2, the 10 MΩ feedback resistance converts the 1 nA–100 nA input current into approximately 10 mV–1 V. Similarly, for Range 3, the 100 kΩ feedback resistance converts the 100 nA–10 µA input current into approximately 10 mV–1 V. The transimpedance output is amplified by an inverting amplifier with a nominal gain of −10, implemented using an OPA2111 dual operational amplifier from Burr Brown (Tucson, AZ, USA). Consequently, the complete CVCU provides an output voltage of approximately 0 V–10 V for Range 1 and 0.1 V–10 V for Ranges 2 and 3. In addition to providing the required voltage gain, the inverting amplifier reverses the polarity of the negative transimpedance output and generates a positive voltage for the subsequent filtering and analog-to-digital conversion stages (see Section 3.2).

Special measures have been adopted in the design of the CVCU to minimize leakage on the printed circuit board (PCB), and therefore ensure high measurement accuracy. A combination of guarding and air wiring, together with proper PCB layout design, has been applied. The input signal is wired through air directly to the inverting input of the AD549J amplifier, thus minimizing the PCB stray capacitance which can compromise the measurement accuracy. In addition, each input terminal of AD549J is guarded with a PCB trace connected to the ground potential, thus isolating the possible leakage path. The feedback components of the transimpedance stage are placed close to the amplifier terminals to minimize parasitic interconnections and unwanted capacitive coupling. Finally, the PCB with the complete CVCU is enclosed in a metal container to minimize external noise interference.

### 3.2. Control and Data Processing Unit (CDPU)

**Figure 4:**
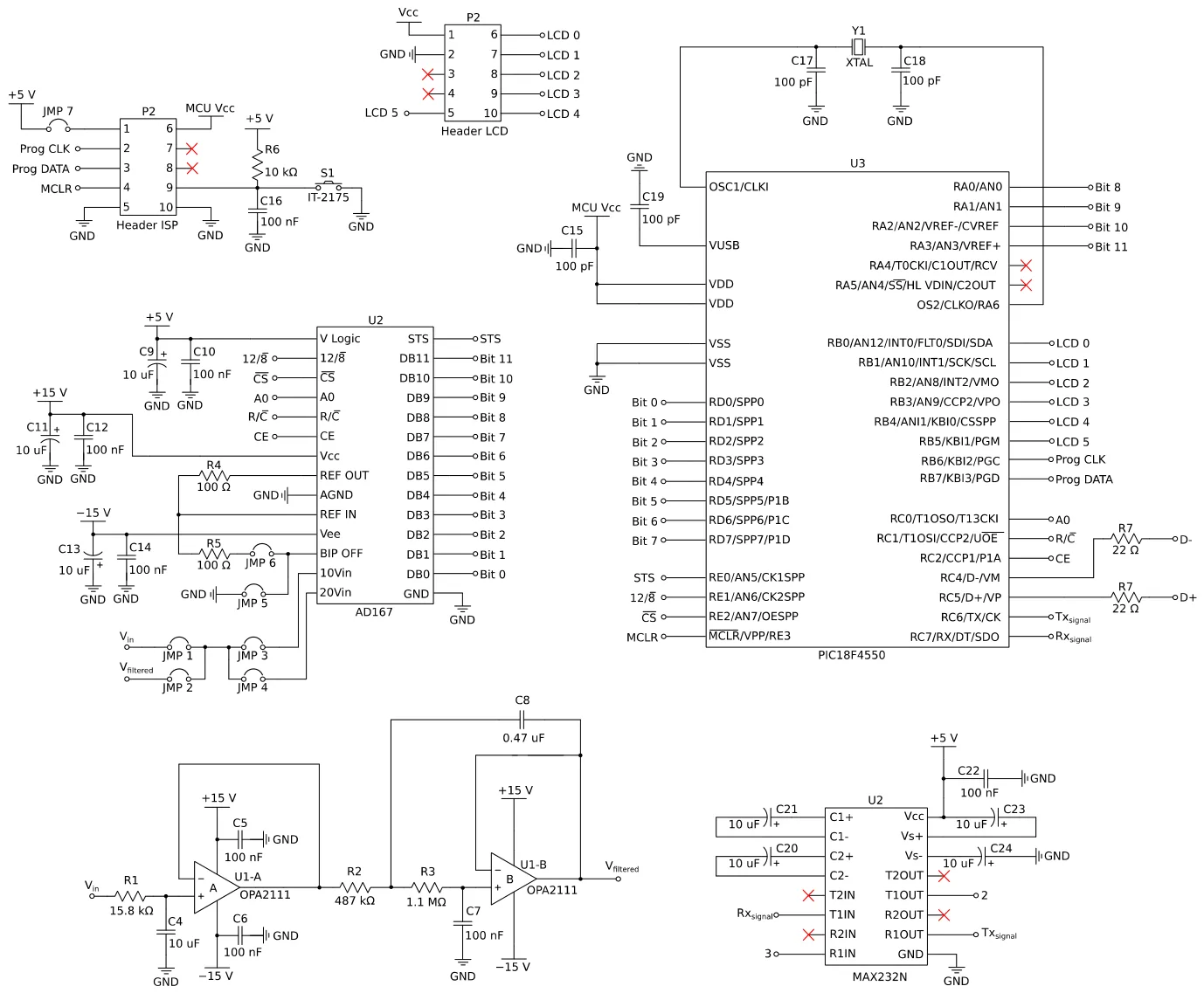
Control and data processing unit.

The Control and Data Processing Unit (CDPU) performs four tasks: (i) Automatic selection of the measurement range, (ii) Digitization of the voltage generated at the output of CVCU, (iii) Transfer of digitized data to the PC, and (iv) Display of measured current on a liquid crystal display (LCD). It consists of a microcontroller (MCU), an analog-to-digital converter (ADC), a low pass filter, and a serial communication interface. The complete schematic of the CDPU is illustrated in Figure 4.

The MCU is the core element of the CDPU, and it executes the firmware responsible for implementing the aforementioned tasks. A general-purpose 8-bit MCU PIC18F4550 from Microchip (Chandler, AZ, USA) has been selected for this purpose. This MCU has a program memory of 32 kB, which is sufficient for implementing the firmware for controlling the current measurement procedure. Its main advantage over other similar MCUs is that it supports both RS232 and USB connections.

When the electrometer is started, by sending a command from the PC over a serial interface, the MCU determines the appropriate measurement range by successively activating the resistors in the variable gain transimpedance amplifier. For each current range, the voltage at the CVCU’s output is digitized and the obtained result is processed in the MCU to determine if the measured voltage is valid. For Range 1, a voltage from 0 V to 10 V is considered as valid. For Ranges 2 and 3, a voltage from 0.1 V to 10 V is considered as valid. The valid voltage level and the information about the corresponding range are sent to the PC for further processing. If the measured voltage does not belong to any range, the MCU sends an appropriate indication to the PC. These tasks are performed until an appropriate command for stopping the read-out is received from the PC. More details on range switching are given in Section 3.4.

A 12-bit ADC AD1674 from Analog Devices is used for digitizing the voltage from the CVCU. This ADC features parallel output, unipolar or bipolar operation mode, four input voltage ranges (0–10 V and 0–20 V in unipolar mode, and −5 V to +5 V and −10 V to +10 V in bipolar mode), conversion speed of 10 μs, sampling rate of 100 ksps, and on-chip voltage reference. In this case, the ADC is used in unipolar mode configured for input voltage range 0–20 V. The 12 pins for data transfer, and the 5 pins for controlling the ADC, are interfaced to the general-purpose pins of the MCU.

The output voltage of the inverting amplifier is fed to the ADC. Before being processed in the ADC, the signal from the CVCU is filtered to minimize the noise that might have been superimposed on the signal. Filtering is achieved by the third-order active low-pass Butterworth filter, developed with the open-source filter design software tool FilterLab 2.0 from Microchip. The filter is implemented with a dual operational amplifier OPA2111, and its cut-off frequency is set to 1 Hz.

The communication between the MCU and the PC can be established over serial RS-232 interface or USB interface. The RS-232 interface is realized with a standard logic level translator MAX232, and it supports full-duplex communication at the speed of 9600 bit/s, with two data lines (one for data reception and one for data transmission). The USB interface is implemented using the USB controller embedded in the MCU.

### 3.3. Power Supply Unit (PSU)

**Figure 5:**
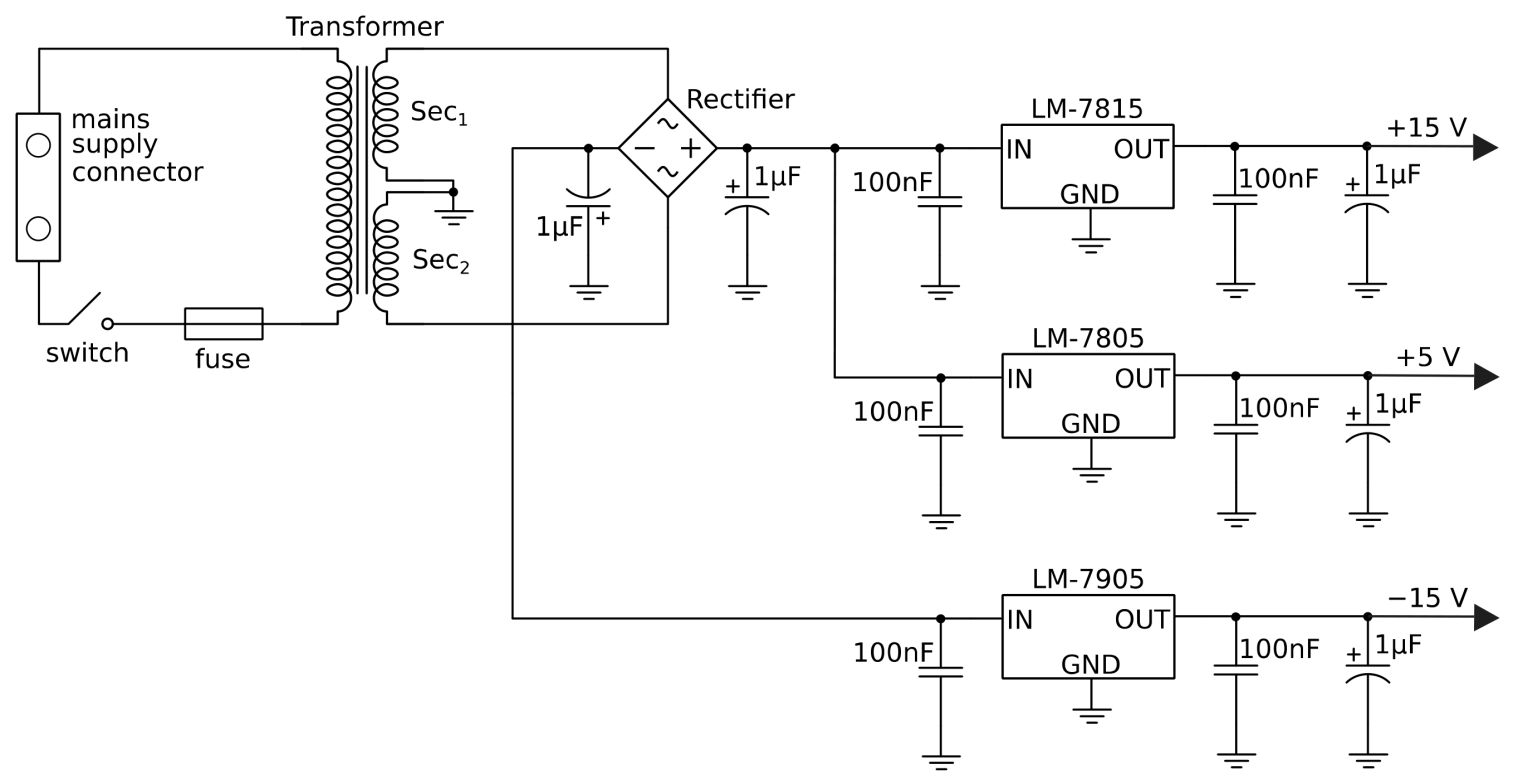
Power supply unit.

The power supply unit (PSU), illustrated in Figure 5, converts the input mains AC voltage into three stabilized DC voltage levels for supplying the electrometer units: +15 V, −15 V, and +5 V. The mains voltage is converted into unregulated voltage by a transformer. Because it was necessary to provide both positive and negative supply voltages, the transformer with two secondary coils has been selected. The voltage from the transformer is rectified by the 4-diode Graetz bridge. For converting the voltage from the rectifier into the stabilized voltages for supplying the electrometer units, three linear voltage regulators have been used: LM7815 (for +15 V voltage), LM7805 (for +5 V voltage), and LM7915 (for −15 V voltage). The +15 V and −15 V are used for supplying the analog components while the +5 V supplies the digital section (MCU).

### 3.4. MCU Firmware

**Figure 6:**
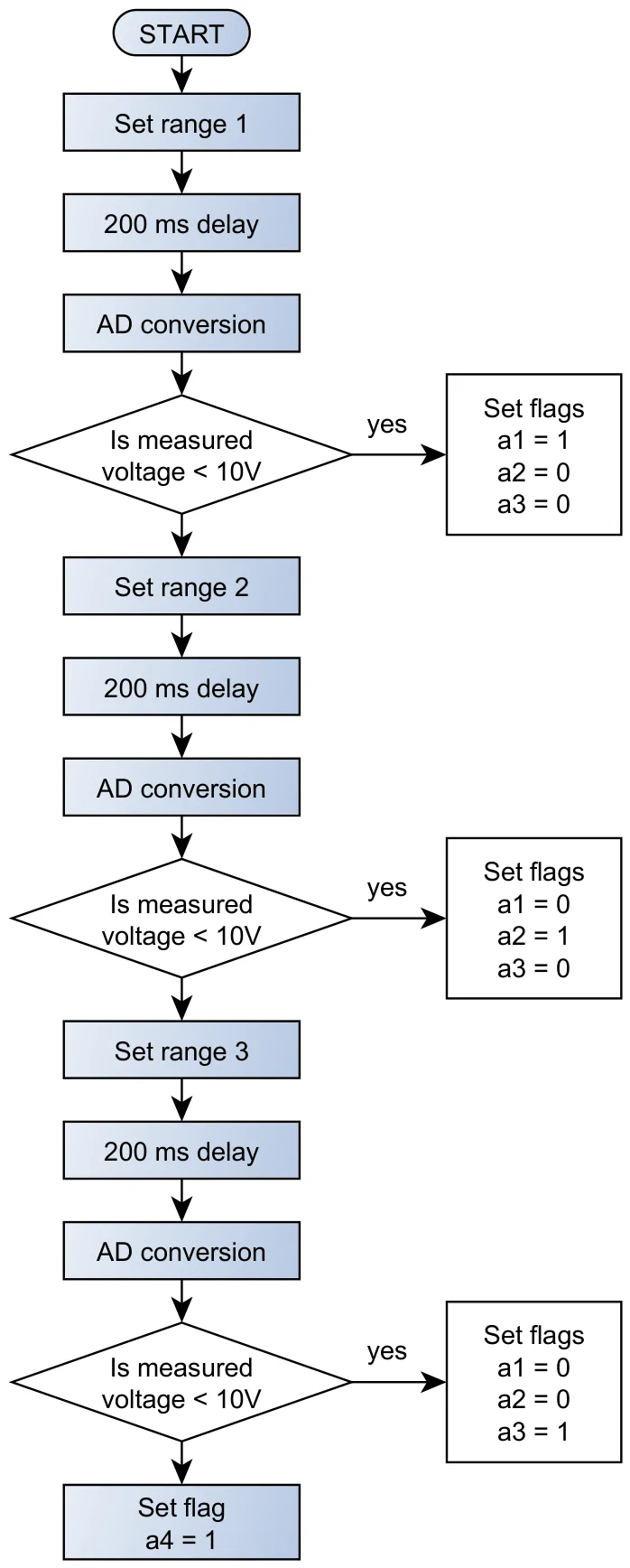
Flow chart for range definition function of MCU.

**Figure 7:**
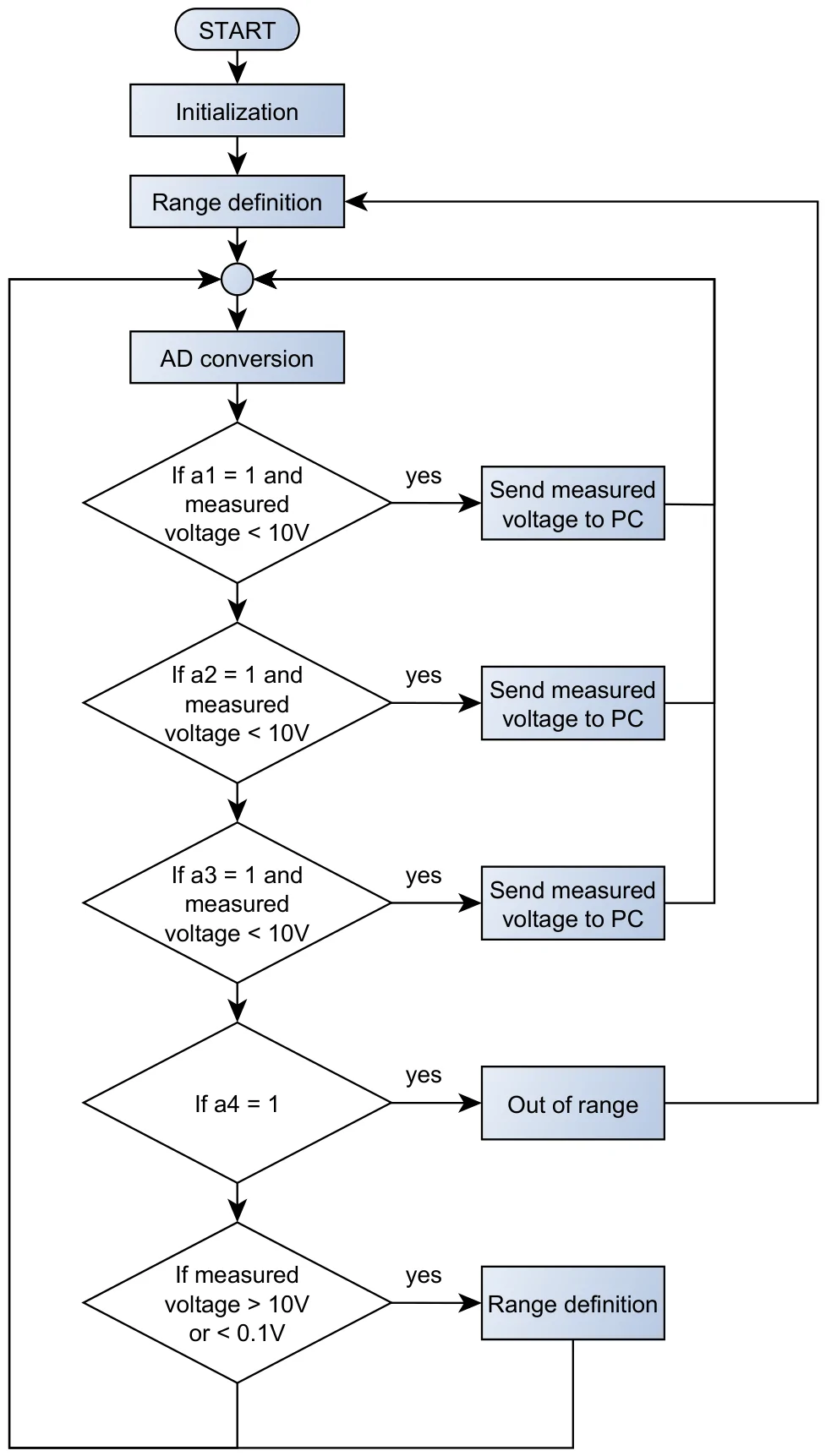
Flow chart for main program of MCU.

The performance of the auto-ranging electrometer depends on the range switching algorithm implemented in the MCU firmware. The range switching algorithm is a sepa-rate function executed within the main program. The rationale for this approach was to ensure the fastest possible response of the electrometer. Figure 6 illustrates the flow chart of the range switching algorithm, and Figure 7 shows the flow chart of the main program. The firmware was developed using the MikroC Pro 6.5.0 development environment [37] from Mikroelektronika, which was also used for programming the MCU.

As shown in Figure 6, the three measurement ranges are successively set by closing the appropriate switch. After closing the switch, a 200 ms delay is established to allow the analog signal to stabilize. The analog-to-digital conversion is then performed and the obtained value is assessed. If the measured value is less than 10 V, a corresponding software flag is set. Due to specific hardware construction of the transimpedance stage, as described previously, only one flag can be set at a time. If the measured voltage exceeds 10 V for all three ranges, the input current is considered to exceed 10 µA, and the corresponding out-of-range flag is set.

The range definition function is called from the main program when a change in the input current range is detected. To ensure fast response, the range definition function is not executed during each measurement cycle but only when the measured value exceeds the 0.1 V and 10 V threshold levels. When the measured voltage remains within the valid interval of the currently selected range, the electrometer continues measurement without re-executing the complete range definition procedure. Because the range switching algorithm is deterministic and involves only a small number of threshold comparisons, ADC measurements, and digital control of analog switches, no numerical optimization or iterative procedure is required.

### 3.5. PC Application Software

A PC application program has been developed for the real-time monitoring of the electrometer read-out and data acquisition. The application software has been developed using the C# programming language and the Visual Studio 2015 (Community edition). The main functionalities of the application software are:

- Starting and stopping the electrometer read-out by issuing adequate commands;
- Converting the measured voltage into the corresponding current, based on the transimpedance relation and calibration function;
- Determining the elapsed time between two successive measurements on the basis of the data obtained from the PC’s real-time clock;
- Storing the measured current and the corresponding elapsed time values in appropriate files on the PC hard drive;
- Displaying the measured current values, as well as dose rate and total dose calculated from current values, on the PC monitor in real time.

## 4. Electrometer Testing

In this section, the evaluation results of the developed electrometer are presented. The electrometer was initially calibrated in a laboratory using a high-performance source-measuring unit as a reference instrument. Experimental testing was done with commercial PIN photodiodes and gamma irradiation. The performance metrics are compared with custom-designed electrometers published in the literature and several commercial dosimetric electrometers.

### 4.1. Calibration Procedure

For the initial testing and calibration of the developed electrometer, a high-precision source-measuring unit Keithley 2636A (Solon, OH, USA) was used. This instrument has two channels for simultaneous current/voltage sourcing and measurement over a wide range, and supports PC-compatible interface for control and data acquisition. To evaluate the electrometer’s performance, a predefined set of reference currents was generated by Keithley 2636A and applied to the electrometer input. The electrometer readings were recorded and compared with the reference current values. The electrometer’s performance was assessed in terms of accuracy, resolution, response time, and repeatability.

**Figure 8:**
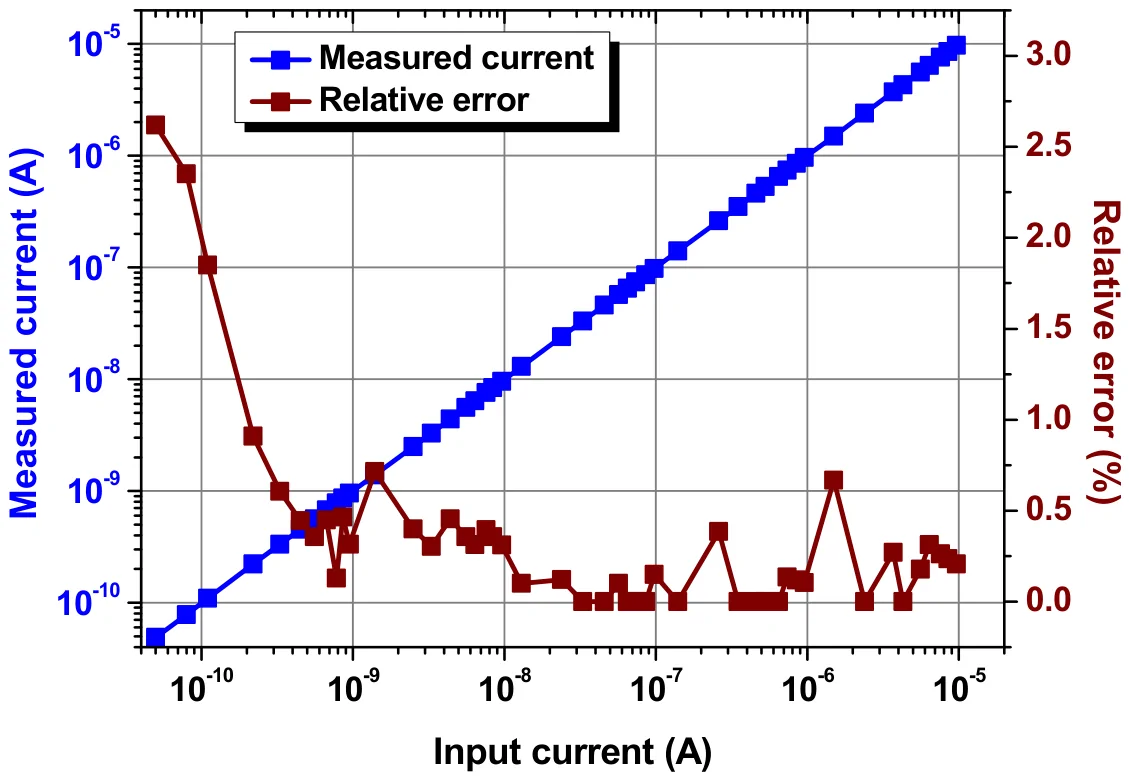
Electrometer calibration curve and relative error.

The primary performance metric is the measurement accuracy, expressed in terms of the relative difference between the electrometer reading and the corresponding reference current. Figure 8 illustrates the resulting calibration curve, showing the relationship between the applied input reference current and the current measured by the electrometer. The corresponding relative error between the measured and reference current values is also shown in Figure 8.

The obtained results indicate that the electrometer is capable of measuring currents from 50 pA to 100 pA with relative error less than 2.5%, and from 100 pA to 10 μA with relative error less than 1%. The developed system can measure even the currents below 50 pA (down to 10 pA) but with relative error up to 10%. These results are comparable with custom-made electrometers reported in the literature [29,34,36], as discussed in more detail in Section 4.3.

As a result of the applied range switching algorithm, the system achieves a typical response time of approximately 0.2 s across all measurement ranges. This represents a ninefold improvement compared with our previous solution, which had a response time of approximately 1.8 s [26].

The reduced response time improves the temporal resolution of dose measurements, enabling smaller dose changes to be detected. It also improves dose rate measurement by reducing the time interval between consecutive measurements, allowing faster variations in the dose rate to be captured. In addition to response time, the measurement resolution depends on the selected current range. The pA range provides a typical resolution of 1 pA, while the nA and μA ranges provide resolutions of 100 pA and 1 nA, respectively.

Finally, the repeatability of the measurements was evaluated by applying different input currents to the electrometer and recording 100 consecutive measurements for each input current level. For all tested input currents, the standard deviation of the measured values was below 0.5%, demonstrating good measurement repeatability.

### 4.2. Irradiation Experiment with PIN Photodiodes

In order to evaluate the applicability of the realized electrometer in current-mode dosimetric applications, it has been employed for measuring the direct current response of commercial PIN photodiodes exposed to gamma radiation. Four commercial PIN photodiodes were chosen for the experiment, and their current response as a function of dose rate and irradiation time was measured. The selected PIN photodiodes are BVP23NF [38], BPW34 [39], PS100-6-CER2 PIN [40], and S1223 [41]. All investigated PIN photodiodes were unbiased during irradiation.

**Figure 9:**
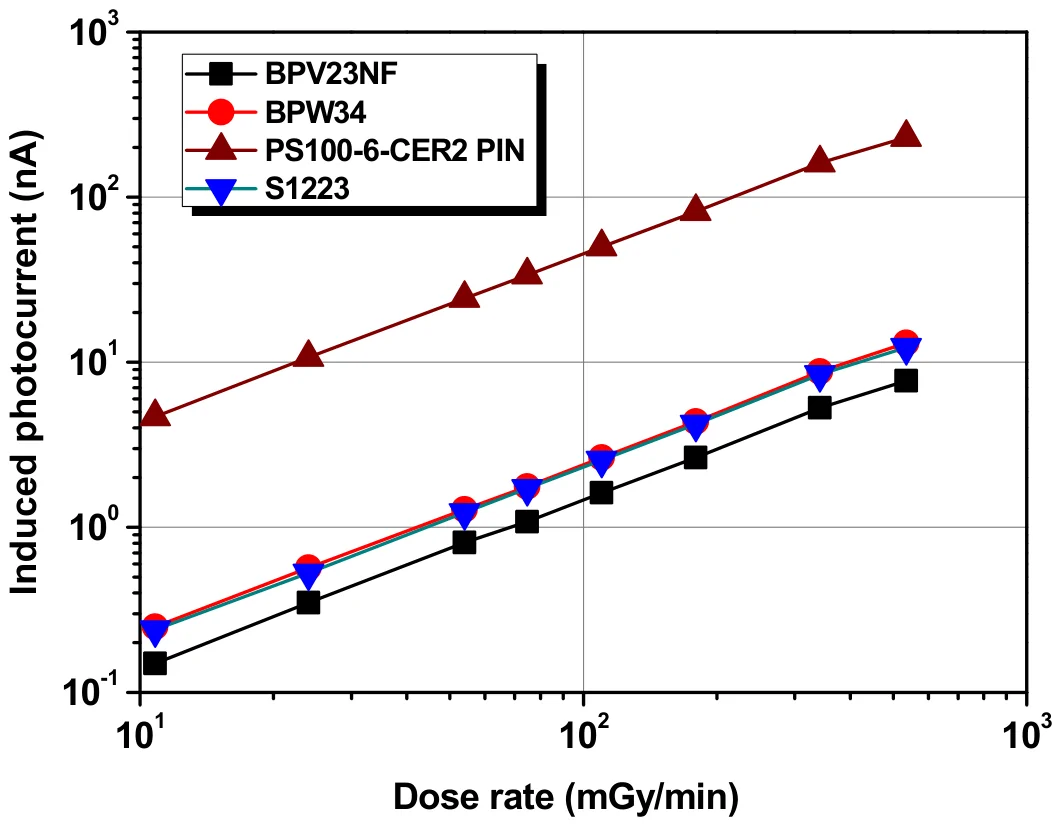
Photocurrent versus dose rate.

**Figure 10:**
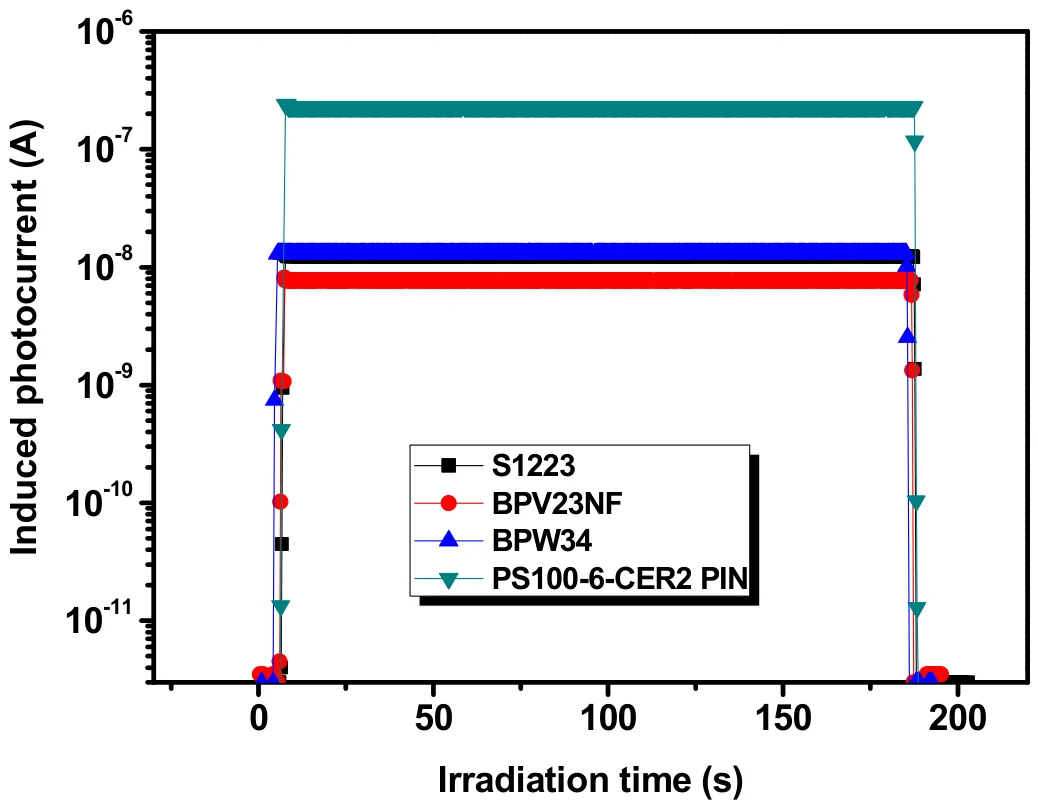
Photocurrent versus irradiation time for dose rate of 34 Gy/h.

The irradiation experiment was performed at the Institute of Nuclear Sciences “Vinča”, Vinča, Belgrade, Serbia. The samples were irradiated by a constant radiation field obtained from a ^60^Co gamma ray source, at room temperature (25 °C). The photodiodes were isolated together with the radiation source in a special irradiation chamber, while the measurement equipment (electrometer and PC) was located in a separate room. The connection between the test samples and the electrometer was achieved through a 5 m coaxial cable, while the interface between the electrometer and the PC was established over an RS232 connection. The variation of the dose rate was accomplished by varying the distance between the samples and the gamma radiation source. Obtained results are illustrated in Figure 9 and Figure 10.

Figure 9 shows the relation between the dose rate and the induced photocurrent. For each dose rate, the PIN photodiodes were irradiated for 3 min and the readings were then averaged to obtain a mean photocurrent value for each dose rate. A good linearity between the dose rate and the induced photocurrent, with correlation coefficient R^2^ > 0.99, was observed for all tested samples, within the range of dose rates 0.9 Gy/h–34 Gy/h. At a given dose rate, the value of induced current was different among the investigated photodiodes, which is attributed to different sensitivities resulting from different sensitive areas. As can be seen, the largest current was measured for PS100-6-CER2 PIN, which has the sensitive area of 100 mm^2^. According to the results in Figure 9, the investigated photodiode PS-100-6-CER2 PIN has the sensitivity of 8.66 nA·Gy^−1^·h, while other PIN photodiodes (all with sensitive areas below 10 mm^2^) have the sensitivities of several hundred pA·Gy^−1^·h. These results indicate that the sensitivity to radiation is proportional to the photodiode’s active area. It can be also observed that PS100-6-CER2 PIN could detect even lower currents, but this was not verified due to technical limitations.

All investigated PIN photodiodes produced stable current response for each dose rate. Figure 10 illustrates only the current response as a function of irradiation time for the dose rate of 34 Gy/h. The radiation-induced photocurrent was significantly greater than the photodiodes’ dark current, and a sudden fall of the photocurrent occurred after the irradiation was stopped. Therefore, when the dose rate is constant over a time interval τ and its value is determined from the measured photocurrent, the total dose absorbed during a time interval τ can be calculated as the product of the dose rate and τ. For the performed experiment, the total absorbed dose per photodiode was up to 33 Gy.

### 4.3. Comparison with Commercial and Custom-Made Electrometers

The main requirements for dosimetric electrometers include a wide dynamic range, high resolution, high accuracy (i.e., low relative error), stable readout, and fast response (i.e., low response time). Conventionally, advanced commercial dosimetric electrometers are calibrated to provide dose rate and absorbed dose measurements for specific radiation sensors based on the measured current or charge. To provide a realistic assessment of the capabilities of the developed electrometer, its technical characteristics are compared with the specifications of several commercial dosimetric electrometers (Table 3) and custom-designed solutions (Table 4).

As can be observed from the data provided in Table 3, the developed electrometer is comparable with the three selected commercial electrometers in terms of measurement range, response time, and repeatability. Based on data given in Table 4, it can be seen that the developed electrometer has comparable performance to that of other similar custom-designed electrometers reported in the literature. However, the proposed electrometer still has some limitations: (i) Does not have integrated bias supply; (ii) has worse resolution than the commercial counterparts; (iii) cannot measure very low currents down to 1 pA or below; and (iv) has not been calibrated to display the total dose. These drawbacks will be addressed in the future upgrades to obtain a more competitive electrometer for general-purpose dosimetric applications.

## 5. Conclusions

An auto-ranging electrometer, supporting direct current measurements from 50 pA to 10 µA with a relative error below 2.5% and nominal response time of 0.2 s, has been designed and implemented. The electrometer was built using commercially available components and calibrated with a high-precision source-measuring unit Keithley 2636A. In order to evaluate the dosimetric performance of the realized electrometer, it was applied for measuring the current response of four commercial PIN photodiodes under gamma radiation exposure from a Co-60 source. The obtained results demonstrate that the developed electrometer provides good performance in terms of measurement accuracy, readout stability, and response time. A comparison with commercial dosimetric electrometers further indicates that the developed system has very satisfactory performance for general purpose applications. The proposed electrometer is also competitive with similar solutions proposed in the literature. As future work, our goal is to enhance the proposed design in order to ensure its tolerance to radiation-induced effects such as soft errors. With radiation tolerance features, the electrometer would be applicable in harsh radiation conditions.

## Figures and Tables

**Table 1:** Current ranges for the developed electrometer.

Measurement Range	Current Range
Range 1	<1 nA
Range 2	1 nA–100 nA
Range 3	100 nA–10 μA

**Table 2:** Transimpedance gain for each range.

Current Measurement Range	Feedback Resistance (MΩ)
Range 1 (<1 nA)	1000
Range 2 (1 nA–100 nA)	10
Range 3 (100 nA–10 μA)	0.1

**Table 3:** Comparison of proposed electrometer and commercial dosimetric electrometers.

	This Work	MAX-4000 [42]	Unidos Tango [43]	Dose-X [44]
Current range	50 pA–10 µA	0.001 pA–500 µA	400 fA–2.6 µA	400 fA–24 µA
Resolution	1 pA–1 nA	1 fA–1 pA	1 fA	0.1 fA
Relative error	<2.5%	0.06%	0.5%	0.25%
Repeatability	0.5%	0.1%	0.25%	0.2%
Response time	0.2 s	<0.5 s	<1.5 s	<1.5 s
Integrated bias source	No	Programmable up to 450 V	Programmable up to 400 V	Programmable up to 500 V
PC interface	RS232 and USB	RS232	USB, LAN, WLAN	Ethernet, Wi-Fi, USB

**Table 4:** Comparison of proposed electrometer and custom-designed electrometers reported in literature.

	This Work	[29]	[34]	[36]
Current range	50 pA–10 µA	10 pA–100 nA	1 fA–5 µA	7 fA–20 µA
Resolution	1 pA–1 nA	-	1 fA	1 fA–10 pA
Relative error	<2.5%	<1.4%	1%	<4%
Used radiation sensor	PIN Photodiodes	Ionization chamber	Ionization chamber	Ionization chamber
Implementation	Commercial components	Commercial components	Custom 350 nm ASIC	Custom 130 nm ASIC

## Data Availability

The experimental data can be shared upon request.

## Abbreviations

The following abbreviations are used in this manuscript:

ADC	Analog-to-Digital Converter
ASIC	Application-Specific Integrated Circuit
CDPU	Control and Data Processing Unit
CMOS	Complementary Metal Oxide Semiconductor
CVCU	Current-to-Voltage Conversion Unit
LCD	Liquid Crystal Display
MCU	Microcontroller Unit
PC	Personal Computer
PCB	Printed Circuit Board

## References

[1] Asensio L.J., Carvajal M.A., Lopez-Villanueva J.A., Vilches M., Lallena A.M., Palma A.J. (2006). Evaluation of Low-Cost Commercial MOSFET as Radiation Dosimeter. Sens. Actuators A Phys..

[2] Poussibet-Garrido A., Escobedo P., Guirado D., Ristic G.S., Palma A.J., Carvajal M.A. (2023). Batteryless NFC Dosimeter Tag for Ionizing Radiation Based on Commercial MOSFET. Sens. Actuators A Phys..

[3] Moreno-Perez J.A., Ruiz-García I., Duane R., Martín-Holgado P., Morvaj L., Vasovic N., Hajdas W., Morilla Y., Carvajal M.A. (2026). Characterization of a 100 nm RADFET as a Proton Beam Detector. Sensors.

[4] Ristic G.S., Ilic S.D., Andjelkovic M.S., Duane R., Palma A.J., Lalena A.M., Krstic M.D., Jaksic A.B. (2022). Sensitivity and Fading of Irradiated RADFETs with Different Gate Voltages. Nucl. Instrum. Methods Phys. Res. Sect. A Accel. Spectrometers Detect. Assoc. Equip..

[5] Andjelkovic M., Simevski A., Chen J., Schrape O., Stamenkovic Z., Krstic M., Ilic S., Ristic G., Jaksic A., Vasovic N. Design of Radiation Hardened RADFET Readout System for Space Applications. Proceedings of the 2020 23rd Euromicro Conference on Digital System Design (DSD).

[6] Marjanovic M., Ilić S.D., Veljković S., Mitrović N., Gurer U., Yilmaz O., Kahraman A., Aktag A., Karacali H., Budak E. (2025). The SPICE Modeling of a Radiation Sensor Based on a MOSFET with a Dielectric HfO_2_/SiO_2_ Double Layer. Sensors.

[7] Ristic G.S., Ilić S.D., Veljković S., Jevtić A.S., Dimitrijević S., Palma A.J., Stanković S., Andjelković M.S. (2022). Commercial P-Channel Power VDMOSFET as X-Ray Dosimeter. Electronics.

[8] Pejovic M.M., Pejovic S.M. (2018). VDMOSFET as a Prospective Dosimeter for Radiotherapy. Appl. Radiat. Isot..

[9] De Meyere W., Shanbhag A., Menicucci A. (2024). Floating Gate Dosimeter Characterization for Space. IEEE Trans. Nucl. Sci..

[10] Daros T., Cábia N.C., Piteira J., Schneider M.C. (2025). Design, Modeling, and Characterization of a Floating Gate Dosimeter in Standard CMOS Technology for Sensor Reuse. IEEE Trans. Nucl. Sci..

[11] Ilic S., Jevtic A., Stankovic S., Ristic G. (2020). Floating-Gate MOS Transistor with Dynamic Biasing as a Radiation Sensor. Sensors.

[12] Ruiz-Garcia I., Moreno-Pérez J., Valdivieso P., Escobedo P., Palma A., Vila R., Carvajal M. (2025). High Dose-Rate Gamma Radiation Response of Commercial Off-the-Shelf Diodes. Radiat. Phys. Chem..

[13] Goncalves J.A.C., Mangiarotti A., Antonio P.L., Caldas L.V.E., Bueno C.C. (2024). Dosimetric Parameters and Radiation Tolerance of Epitaxial Diodes for Diagnostic Radiology and Computed Tomographic Beams. Radiat. Phys. Chem..

[14] Amarjit S.S. (2007). In-vivo Radiation Diode Dosimetry for Therapeutic Photon Beams. Ph.D. Thesis.

[15] Andjelkovic M., Ristic G.S. (2015). Current Mode Response of Phototransistors to Gamma Radiation. Radiat. Meas..

[16] Magalhaes C.M.S., Sobrinho M., Souza D., Filho J.A., da Silva E., Santos L. (2012). A Novel Dosimetry System for Computed Tomography Using Phototransistors. Radiat. Meas..

[17] Roman-Raya J., Ruiz-García I., Escobedo P., Palma A.J., Guirado D., Carvajal M.A. (2020). Light-Dependent Resistors as Dosimetric Sensors in Radiotherapy. Sensors.

[18] Aoyama T., Koyama S., Kawaura C. (2002). An In-phantom Dosimeter System Using PIN Silicon Photodiode Radiation Sensors for Measuring Organ Doses in X-ray CT and Other Diagnostic Radiology. Med. Phys..

[19] Ferreira D.C., Napolitano C.M., Gonçalves J.A.C., Bueno C.C. Use of a PIN Photodiode for Dosimetric Purposes in Radiation Processing. Proceedings of the 2009 International Nuclear Atlantic Conference (INAC 2009).

[20] Paschoal C.M.M., Souza D.N., Santos L.A.P. (2011). Characterization of Three Photodetector Types for Computed Tomography Dosimetry. Int. J. Biomed. Biol. Eng..

[21] Silva I.T., Pieri K., Albino L.D., Fontana T.S., dos Santos M.F., Roesler E., Oliveira C.N., Asfora V.K., Gonçalves J.A., Khoury H.J. (2024). Characterization of a Commercial PIN Diode for Radiotherapy Photon Beam Dosimetry. Radiat. Phys. Chem..

[22] Drndarevic V., Jevtic N., Rajovic V., Stankovic S. (2014). Smart Ionization Chamber for Gamma-Radiation Monitoring. Nucl. Technol. Radiat. Prot..

[23] Angelou C., Patallo I.S., Doherty D., Romano F., Schettino G. (2024). A Review of Diamond Dosimeters in Advanced Radiotherapy Techniques. Med. Phys..

[24] Acharya Y.B. (2006). Logarithmic Current Electrometer Using Commercially Available Current Conveyor AD844. Int. J. Electron..

[25] Khairurrijal, Abdullah M., Suhendi A., Munir M.M., Surachman A. (2007). A Simple Microcontroller-Based Current Electrometer Made from LOG112 and C8051F006 for Measuring Current in Metal-Oxide Semiconductor Devices. Meas. Sci. Technol..

[26] Andjelkovic M., Ristic G. (2013). Feasibility Study of a Current Mode Gamma Radiation Dosimeter based on a Commercial PIN Photodiode and a Custom Made Autoranging Electrometer. Nucl. Technol. Radiat. Prot..

[27] Nazififard M., Suh K.Y., Mahmoudieh A. (2016). Experimental Analysis of a Novel and Low-Cost PIN Photodiode Dosimetry System for Diagnostic Radiology. Rev. Sci. Instrum..

[28] Yang Y., Yu T., Zhang J., Wang W., Gui H., Du P., Liu J. (2018). Design and Evaluation of an Aerosol Electrometer with Low Noise and a Wide Dynamic Range. Sensors.

[29] Bruning F.F., Maia J.M. Low-Cost Electrometer for Use in Ionizing Radiation Metrology. Proceedings of the IX Latin American Congress on Biomedical Engineering and XXVIII Brazilian Congress on Biomedical Engineering (CLAIB 2022 & CBEB 2022).

[30] Chen C., Zeng W., Yang Z., Hou S., Liang J., Liu H. (2025). Development of a Current Measurement System for 4πγ Ionization Chamber. Appl. Radiat. Isot..

[31] Biyik R., Yalcin T., Alacayir O. (2026). A Portable Femtoampere-Sensitive Readout System for a High-Pressure Argon Ionization Chamber. IEEE Trans. Instrum. Meas..

[32] Temirzhanov A.A., Issatov A., Zholdybayev T.K., Sadykov B.M., Mendibayev K. (2026). Low Cost Nanoamperemeter Based on Switched Integrator Circuit. Interactions.

[33] Voulgari E., Noy M., Anghinolfi F., Perrin D., Krummenacher F., Kayal M. (2016). A Front-end ASIC for Ionising Radiation Monitoring with Femtoamp Capabilities. J. Instrum..

[34] Voulgari E., Noy M., Anghinolfi F., Krummenacher F., Kayal M. (2018). Utopia: A Nine Decade Femtoampere Sensitivity Current Digitizer and its Application in Ionizing Radiation Monitoring. IEEE Trans. Nucl. Sci..

[35] Mohanan S., Boukabache H., Perrin D., Pfeiffer U.R. (2021). Comparative Analysis of Ultra-Low Current Measurement Topologies with Implementation in 130 nm Technology. IEEE Access.

[36] Mohanan S., Boukabache H.E., Cruchet V., Perrin D. (2022). An Ultra-Low Current Measurement Mixed-Signal ASIC for Radiation Monitoring Using Ionizing Chambers. IEEE Sens. J..

[37] MikroC Development Environment. http://www.mikroe.com.

[38] https://www.vishay.com/search/?searchChoice=part&query=BPV23NF.

[39] https://www.vishay.com/search/?searchChoice=part&query=BPW34.

[40] https://pdf.datasheet.support/76c29028/first-sensor.com/PS100-6-CER2PIN.html.

[41] https://www.hamamatsu.com/eu/en/product/optical-sensors/photodiodes/si-photodiodes/S1223-01.html.

[42] https://www.standardimaging.com/electrometers/max-4000-plus-electrometer.

[43] https://www.ptwdosimetry.com/en/products/unidos-tango-and-unidos-romeo-reference-class-electrometers.

[44] https://www.iba-dosimetry.com/product/dose-x.

